# Normalized Lactate Load Is Associated with Development of Acute Kidney Injury in Patients Who Underwent Cardiopulmonary Bypass Surgery

**DOI:** 10.1371/journal.pone.0120466

**Published:** 2015-03-30

**Authors:** Zhongheng Zhang, Hongying Ni

**Affiliations:** Department of Critical Care Medicine, Jinhua Municipal Central Hospital, Jinhua Hospital of Zhejiang University, Zhejiang, P.R.China; Bambino Gesù Children's Hospital, ITALY

## Abstract

**Background and Objective:**

Cardiac surgery associated acute kidney injury is a major postoperative complication and has long been associated with adverse outcomes. However, the association of lactate and AKI has not been well established. The study aimed to explore the association of normalized lactate load with AKI in patients undergoing cardiac surgery.

**Methods:**

This was a prospective observational cohort study conducted in a 47-bed ICU of a tertiary academic teaching hospital from July 2012 to January 2014. All patients undergoing cardiopulmonary bypass surgery were included. Normalized lactate load (L) was calculated by the equation: $L=\frac{1}{2\times \left(t_{n}-t_{0}\right)}\sum_{i=0}^{n}\left(v_{i}+v_{i+1}\right)\times \left(t_{i+1}-t_{i}\right)$, where t_i_ was time point for lactate measurement and v_i_ was the value of lactate. L was transformed by natural log (L_ln_) to improve its normality. Logistic regression model was fitted by using stepwise method. Scale of L_ln_ was examined by using fractional polynomial approach and potential interaction terms were explored.

**Results:**

A total of 117 patients were included during study period, including 17 AKI patients and 100 non-AKI patients. In univariate analysis L_ln_ was significantly higher in AKI as compared with non-AKI group (1.43±0.38 vs 1.01±0.45, p = 0.0005). After stepwise selection of covariates, the main effect logistic model contained variables of L_ln_ (odds ratio: 11.1, 95% CI: 1.22–101.6), gender, age, baseline serum creatinine and fluid balance on day 0. Although the two-term fractional polynomial model was the best-fitted model, it was not significantly different from the linear model (Deviance difference = 6.09, p = 0.107). There was no significant interaction term between L_ln_ and other variables in the main effect model.

**Conclusions:**

Our study demonstrates that L_ln_ is independently associated with postoperative AKI in patients undergoing CPB. There is no significant interaction with early postoperative fluid balance.

## Introduction

Acute kidney injury (AKI) is a serious complication in patients undergoing cardiopulmonary bypass (CPB) surgery [1]. Cardiac surgery is the second leading cause of AKI in the intensive care unit (ICU), just following sepsis [2]. Due to the different definitions of AKI and surgical types, its incidence is reported to be between 10% and 40% [3–5]. Although the pathophysiology of AKI following cardiac surgery is largely unknown, the etiology is likely to be multifactorial and is associated with baseline renal function, intraoperative and early postoperative management. Given this pathophysiologic complexity, no single strategy exists for the prevention of AKI.

One purpose in AKI research is to identify potential biomarkers for the prediction of AKI after insult from cardiac surgery. Extensive studies have been conducted in this field and these biomarkers include but not limited to Cyctatin C, neutrophil gelatinase-associated lipocalin (NGAL), urine IL-18 and B-type natriuretic peptide (BNP) [6–9]. One potential pathway of AKI is hypoperfusion and ischemia during cardiac surgery [10,11]. We proposed that biomarkers associated with hypoperfusion and ischemia may be a good candidate for the prediction of AKI development after cardiac surgery.

Lactate is a well-known biomarker of tissue hypoxia (type A hyperlactatemia). Type B hyperlactatemia was not directly associated with tissue hypoxia [12]. It can be further sub-divided depending on whether it is caused by underlying disease (B1), drugs and toxins (B2) or inborn errors of metabolism (B3) [13]. In type B hyperlactatemia the serum lactate:piruvate ratio remains near normal (differently from type A hyperlactatemia [14]. Both type A and type B hyperlactatemia are frequently observed in patients undergoing cardiac surgery [15,16]. Fluid infusion with Hartmann’s solution may also transiently result in hyperlactatemia until it is metabolized by the liver especially when this fluid is used to prime the CPB circuit [17]. However, lactate has never been systematically studied for its association with AKI in CPB patients. Furthermore, previous studies have only investigated the impact of lactate and its change on outcomes [18–20]. Since lactate is a sensitive biomarker of global and regional hypoperfusion [21], lactate can be used as a marker of ongoing hypoperfusion that may contribute to ongoing development of AKI. Both the magnitude and time interval of hyperlactatemia should be considered. In this regard, we coined a new term “normalized lactate load” to account for the magnitude and time of lactate in the present study. We hypothesized that normalized lactate load was associated with AKI after cardiac surgery.

## Methods

This was a prospective observational cohort study conducted in a 47-bed ICU of a tertiary academic teaching hospital from July 2012 to January 2014. The study was approved by the ethics committee of Jinhua municipal central hospital. Informed consent was waived due to observational nature of the study. Patient records/information was anonymized and de-identified prior to analysis.

All patients undergoing cardiac surgery with CPB were potentially eligible to the present study. In our institution, all CPB patients were entered into ICU for monitoring and postoperative care. Exclusion criteria included: pediatric patients (age<15 years old); patients with severe complications who were expected to survive < 24 hours (e.g. massive hemarrhage, cardiac temponade, refractory circulatory shock that causes multiple organ failure with SOFA>15); patients with preexisting end stage renal disease requiring intermitant hemodialysis.

On ICU entry the following data were obtained for each participant: age, gender, body weight, serum creatinine, fluid balance on a daily basis, APACHE ǁ score, primary reasons for CPB surgery (e.g. Rheumatic heart disease, Valvular disease, Mitral valve prolapse, Myxoma, Congenital heart disease, Pulmonary venous thrombosis), on pump time, cross-clamp time, microalbuminuria, urinary albumin to creatinine ratio, and serial measurements of lactate. Patients were followed for the whole hospital stay and following data were extracted: length of stay (LOS) in ICU and hospital, duration of mechanical ventilation, postoperative hospital LOS. AKI was defined by an increase of serum creatinine (Scr)×1.5 to the baseline Scr, and Baseline Scr was measured on hospital admission before operation. On the other hand, AKI was also defined when urine output<0.5 ml/kg/min for more than 6 hours [22]. Lactate was measured by using point of care analyzer in the ICU (ABL800 FLEX analyzer, Radiometer). The reference range was less than 2.0 mmol/l, with total variation of less than 20%.

**Fig 1:**
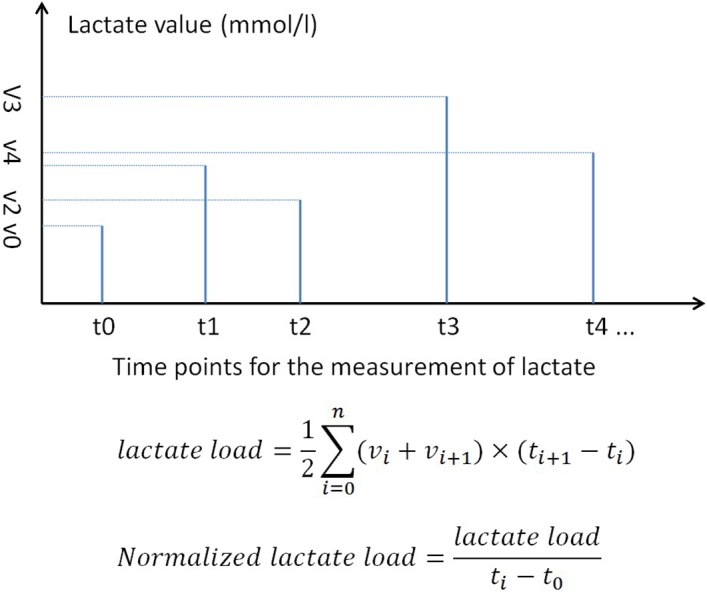
Schematic illustration of the calculation of lactate load and normalized lactate load.

No protocol on the measurement of lactate was made for the sole purpose of this study. In clincal practice, serum lactate was generally measured at 4-hour interval for the first 24 hour after surgery. The treating physician can modify the measurement frequency as needed. A study coordinator not involved in the management of patients was responsible for the recording of lactate and the time when the blood sample was taken. Lactate load was defined as the product of time and lactate value, and normalized lactate load was the lactate load divided by the time (Fig. 1). Lactate load was used to account for cumulative effect of hyperlactatemia in predicting outcome. Our previous work has demonstrated that both lactate level and time influenced clinical outcome [23]. The rationale is that a patient with persistent hyperlactatemia may have worse outcome than those with transient hyperlactatemia, given the magnitude of hyerlactatemia is the same. For instance, if there were three measurements of lactate defined as v1, v2 and v3, with corresponding measurement time of t1, t2 and t3, then the lactate load and normalized lactate load would be given by the equations *lactate load = [(v1+v2)×(t2-t1)+(v2+v3)×(t3-t2)]/2* and normalized lactate load = [(v1+v2)×(t2-t1)+(v2+v3)×(t3-t2)]/[2*(t3-t1)], respectively. Due to expected skewed distribution of normalized lactate load, it was log transformed to improve its normality. The log transformed one is denoted as L_ln_ throughout the manuscript. Lactate load considers both magnitude and time of hyperlactatemia. Other studies considered lactate and time separately, which was subject to the problem of multiple testing [24,25]. However, it may not be valid in the situation when a patient has prolonged measurement of lactate while the other has short time of lactate measurements. For example, patient A has low magnitude of lactate but long time, patient B has high magnitude of lactate but short time of measurement. Their lactate loads can be equal. In this situation we used normalized lactate load to acount for difference in the measurement duration. Another reason for the use of such metrics is that the study is observational in nature and the time point of lactate measurements had not been predefined. Thus it is impossible to calculate lactate clearance at predefined time points such as 4-hour and 6-hour as has been done in other studies.

### Statistical analysis

Data were expressed as mean±SD (standard deviation) or median and interquartile range (IQR) as appropriate. If the variable normalized lactate load was not normally distributed in the dataset, it would be log-transformed to improve its normality. Test for normality was performed by using the syntax *sktest*, which presented a test for normality based on skewness and another based on kurtosis and then combined the two tests into an overall test statistic. Student’s t test or Wilcoxon rank-sum test was performed for comparisons between AKI and non-AKI groups.

Covariates for multivariable logistic regression analysis were chosen by using stepwise selection procedure. Variables that might have potential influences on the relationship between AKI and L_ln_ were all included into the initial model (all variables listed in Table 1). Stepwise forward selection and backward elimination procedure were performed to build the main effect model, because this procedure can provide a fast and effective means to screen a large number of covariates. We chose alpha level of 0.3 and 0.15 for the elimination and entry criteria.

**Table 1:** Comparisons of characteristics of patients with and without AKI.

Variables	Overall (n = 117)	Non-AKI (n = 100)	AKI (n = 17)	P
Age (years)	50.62±12.46	50.46±12.26	51.59±13.94	0.73
Gender (male, percent)	43 (36.8%)	37 (37.0%)	6 (35.3%)	0.893
Body weight (kg)	59.0±12.0	59.0±10.1	58.7±12.0	0.897
APACHE ǁ	11 (8–15)	11 (8–14)	13 (9–15)	0.159
Primary reasons for CPB surgery (n, %)				0.047 by Fisher’s exact test
Rheumatic heart disease	82 (70.09)	73 (73)	9 (52.94)	0.095
Valvular disease	19 (16.24)	15 (15)	4 (23.52)	0.474
Myxoma	5 (4.27)	4 (4)	1 (5.88)	0.551
Mitral valve prolapse	2 (1.71)	0 (0)	2 (11.76)	0.02
Congenital heart disease	8 (6.84)	7 (7)	1 (5.88)	0.484
Pulmonary venous thrombosis	1 (0.85)	1 (1)	0 (0)	1.000
Duration of CPB (minutes)	69.0±28.0	66.6±26.4	82.9±33.8	0.026
Cross-clamp time (minutes)	47.9±22.4	46.2±20.9	57.8±28.3	0.049
Inotrope (n, %)	94 (80.3)	82 (82.0)	12 (70.6)	0.274
Vasopressor (n, %)	37 (31.6)	29 (29.0)	8 (47.1)	0.139
Microalbuminuria	2.05±1.93	1.97±1.99	2.49±1.55	0.307
Urinary albumin to creatinine ratio (g/mol)‡	1.13±0.77	1.02±0.66	1.83±1.01	<0.001
Baseline creatinine (mmol/l)	87.6±25.1	89.0±25.2	75.8±21.3	0.084
Fluid balance				
D0 (ml/24 hr)	751±832	677±828	1319±646	0.0083
D1 (ml/24 hr)	192±825	209±841	61±704	0.545
D2 (ml/24 hr)	-222±1106	-231±1136	-154±896	0.817
D3 (ml/24 hr)	-582±1126	-631±1148	-207±881	0.2211
Hospital LOS (days)	25.4±9.6	25.7±9.1	23.6±9.6	0.5568
Postoperative hospital LOS (days)	14.4±4.6	14.5±4.5	13.4±5.1	0.5526
ICU LOS (days)	4.2±2.2	4.1±2.2	4.4±1.9	0.6585
Duration of mechanical ventilation (hours)	22.2±43.9	22.2±47.0	21.8±13.8	0.9682
Lactate load (mmol•hr/l)‡	4.47±0.57	4.39±0.54	4.92±0.54	0.0003
Normalized lactate load (mmol/l)‡	1.07±0.47	1.01±0.45	1.43±0.38	0.0005

‡ These variables were log-transformed to improve its normality.

After the main effect model was built, we proceeded to examine the scale of L_ln_ in the Logit. The analytic approach was based on fractional polynomials as developed by Royston and coworkers [26]. For simplicity, the power of L_ln_ was restricted to the set of (−2, −1, −0.5, 0, 0.5, 1, 2, 3), where the power of 0 denotes the log of L_ln_. Two terms containing L_ln_ were allowed at most. Implementation of the analytic approach requires fitting 36 models (8 for one term model and $\;\sum_{k\;=\;1}^{7}k$ = 28 two-term models), and the best model is the one with the largest log-likelihood (smallest deviance). After obtaining the best one-term and two term models, the next question is to determine whether either of the two best models is significantly better than the linear model. We used closed test procedure that begins by comparing the best two-term model to the linear model. If this test is not significant, we stop and use the linear model. If the test is significant then the best two-term model is compared to the best one-term model. If this test is not significant then we select the best one-term model, otherwise select the two-term model [27,28].

Interaction terms between L_ln_ and other variables in main effect model were tested for their significance by using likelihood ratio test. Statistically significant terms would be included into the main effect model to build the final model.[29]

Model fit was tested by using Hosmer-Lemeshow method, which examined whether or not the observed event rates match expected event rates in subgroups of the model population. Model discrimination was assessed graphically by using the following plots: receiver-operating characteristic (ROC) curve, a histogram and dot plot of the risk score in the AKI and non-AKI groups.[30]

All statistical analyses were performed by using the software Stata 13 (StataCorp, College Station, Texas 77845 USA). Two-sided p<0.05 was considered to be statistically significant.

## Results

**Fig 2:**
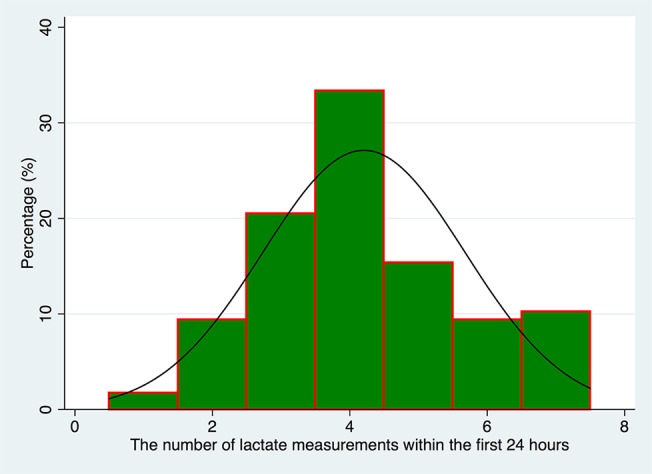
Histogram showing the number of lactate measurements within the first 24 hours.

A total of 117 patients were included during study period, including 17 AKI (12 with RIFLE-R and 5 with RIFLE-I) and 100 non-AKI patients. Characteristics of subjects are shown in Table 1. There was no difference between AKI and non-AKI groups in variables of age, gender, body weight, APACHE ǁ, microalbuminuria and baseline Scr. Patients undergoing CPB because of Mitral valve prolapse appeared to be more likely to develop postoperative AKI (11.76% vs 0; p = 0.02). Patients with longer duration of CPB were more likely to develop AKI (82.9±33.8 vs 66.6±26.4 minutes, p = 0.026). Likewise, longer cross-clamp time was also associated with increased risk of AKI (57.8±28.3 vs 46.2±20.9 minutes, p = 0.049). While microalbuminuria was not significantly associated with postoperative AKI, urinary albumin to creatinine ratio appeared to be higher in AKI patients (1.83±1.01 vs 1.02±0.66 g/mmol, p<0.001). Baseline Scr was not significantly different between AKI and non-AKI groups (89.0±25.2 vs 75.8±21.3 mmol/l, p = 0.084). Except for fluid balance on day0 which is significantly higher in AKI group (1319±646 vs 677±828 ml/24 hour; p = 0.0083), fluid balance on other days were not significantly different between AKI and non-AKI groups. There was no significant difference in clinical outcomes such as hospital and ICU LOS, postoperative hospital LOS and duration of mechanical ventilation. L_ln_ was significantly higher in AKI as compared with non-AKI group (1.43±0.38 vs 1.01±0.45, p = 0.0005). Fig. 2 shows the number of lactate measurements within the first 24 hours.

**Fig 3:**
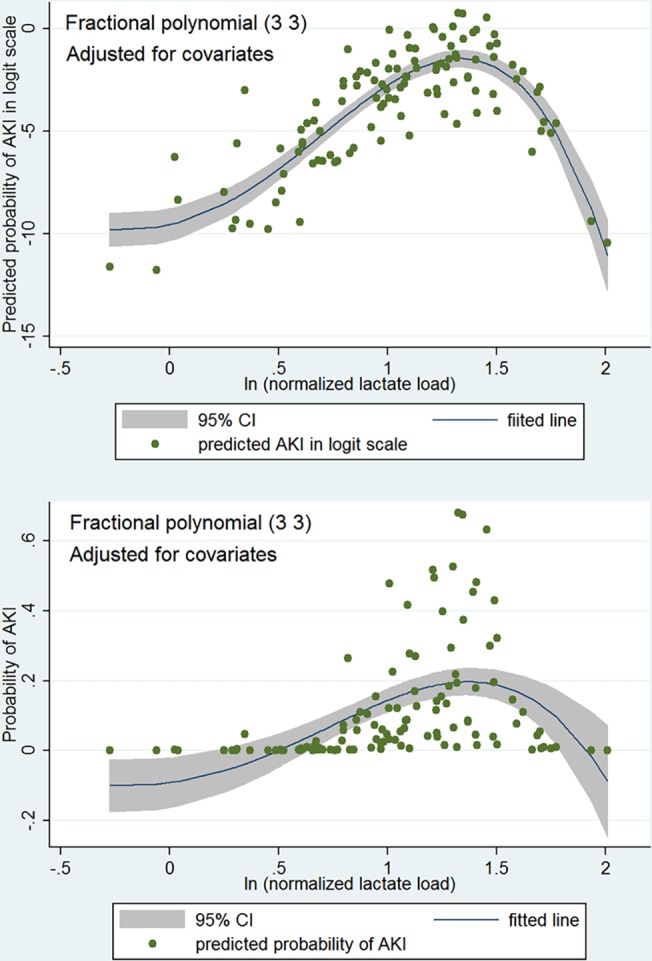
Graphical presentation of the fitted two-term (3 3) fractional polynomial logistic regression model. In the upper panel, the y-axis is in logit scale with the advantage of better distribution for model fit. Y-axis is transformed to the probability of AKI in lower panel which is more comprehensible to subject matter audience. The plots show that the probability of AKI increases progressively with increasing normalized lactate load, reaching its peak at L_ln_≈1.4.

All variables listed in Table 1 were entered into the initial logistic regression model. After stepwise selection, only five variables remained in the model (Table 2). L_ln_ was independently associated with the development of AKI (odds ratio [OR]: 11.1, 95% CI: 1.22–101.6; with each unit increase in log scale). Fluid balance on day 0 was also independently associated with AKI (OR: 1.001, 95% CI: 1.00049–1.0025). The scale of L_ln_ was examined by using fractional polynomial method (Table 3), the result showed that the best two-term (m = 2) model had the smallest deviance (55.395) among all models. The power for L_ln_ in the model was 3 and 3. That is, in the regression model equation, the term L_ln_ assumes the form of L_ln_^3^ and L_ln_^3^ln(L_ln_). In the best two-term model, both terms containing L_ln_ were significantly associated with the development of AKI (Table 4). Fig. 3 is graphical presentation of the fitted two-term (3 3) fractional polynomial logistic regression model. In the upper panel, the y-axis is in logit scale with the advantage of better distribution characteristics for model fit. Y-axis is transformed to the probability of AKI in lower panel which is more comprehensible to subject matter audience. The plots showed that the probability of AKI increased progressively with increasing normalized lactate load, reaching its peak at L_ln_≈1.4. Because there was no statistical difference between two-term model and linear model (deviance difference: 6.09, p = 0.107), we use the linear model to explore potential interaction terms. There was no significant interaction between L_ln_ and other variables, as shown in Table 5 by insignificant likelihood ratio tests (Table 5).

**Table 2:** Main effect model.

Variables	Odds ratio	Standard error	95% CI	p
L_ln_	11.1	12.6	1.22–101.6	0.033
Gender	3.58	3.11	0.65–19.63	0.141
Age	0.92	0.03	0.86–0.99	0.017
Baseline Scr	1.03	0.02	0.98–1.07	0.222
Fluid balance on day0	1.001	0.0005	1.00049–1.0025	0.003

Abbreviations: L_ln_, log transformed normalized lactate load; Scr, serum creatinine.

**Table 3:** Comparisons of fractional polynomial models.

L_ln_	df	Deviance	Deviance difference	P‡	Powers
Not in the model	0	69.016	13.621	0.009	-
Linear	1	61.485	6.090	0.107	1
m = 1	2	58.271	2.876	0.237	−1
m = 2	4	55.395	-	-	3 3

‡P-value from deviance difference comparing reported model with m = 2 model.

Abbreviations: L_ln_, log transformed normalized lactate load.

**Table 4:** Best two-term model with powers of 3 and 3.

Variables	Odds ratio	Standard error	95% CI	P
L_ln_^3^	104.7	214.6	1.89–5814.9	0.023
L_ln_^3^ln(L_ln_)	0.0036	0.0096	0.00002–0.67	0.035
Gender	4.14	3.57	0.76–22.5	0.100
Age	0.92	0.034	0.86–0.99	0.024
Fluid balance on day0	1.001	0.0005	1.0003–1.002	0.008
Baseline Scr	1.02	0.02	0.98–1.06	0.235

Abbreviations: L_ln_, log transformed normalized lactate load; Scr, serum creatinine.

**Table 5:** Potential interaction terms between among variables.

Interaction	Log-likelihood	P for interaction terms	Likelihood ratio test	P for likelihood ratio test
Main effects model	−30.743	-	-	-
L_ln_×gender	−30.159	0.261	1.17	0.2801
L_ln_×age	−30.265	0.350	0.96	0.3283
L_ln_×baseline Scr	−30.700	0.770	0.09	0.7706
L_ln_×fluid balance	−30.345	0.390	0.79	0.3728

Abbreviations: L_ln_, log transformed normalized lactate load; Scr, serum creatinine.

**Fig 4:**
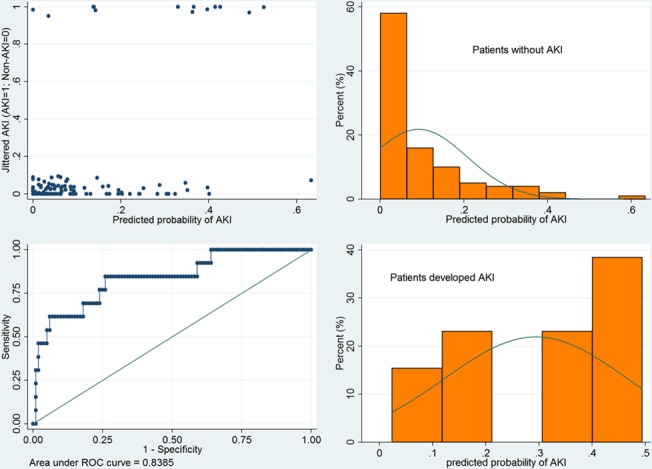
Graphical presentation of model discrimination. Jittered plot shows that non-AKI dots are mostly clustered to the left side and AKI dots are mostly clustered to the right side. ROC curve showed good discriminating power of the model with an area under ROC of 0.84. The histogram shows the distribution of predicted risk score for AKI stratified by observed AKI and non-AKI. In non-AKI group, the distribution of predicted AKI probability skewed to the right; while in AKI group, the distribution of predicted AKI probability skewed to the left. P = 0.2707 for Hosmer-Lemeshow goodness-of-fit test.

Model fit was tested by using Hosmer-Lemeshow goodness-of-fit test, which showed a well-fitted model (P = 0.2707). Graphical presentation of model discrimination is shown in Fig. 4. Jittered plot showed that non-AKI dots were mostly clustered to the left side and AKI dots are mostly clustered to the right side. ROC curve showed excellent discriminating power of the model with an area under ROC of 0.84. The histogram shows the distribution of predicted risk score for AKI stratified by observed AKI and non-AKI. In non-AKI group, the distribution of predicted AKI probability skewed to the right; while in AKI group, the distribution of predicted AKI probability skewed to the left.

**Fig 5:**
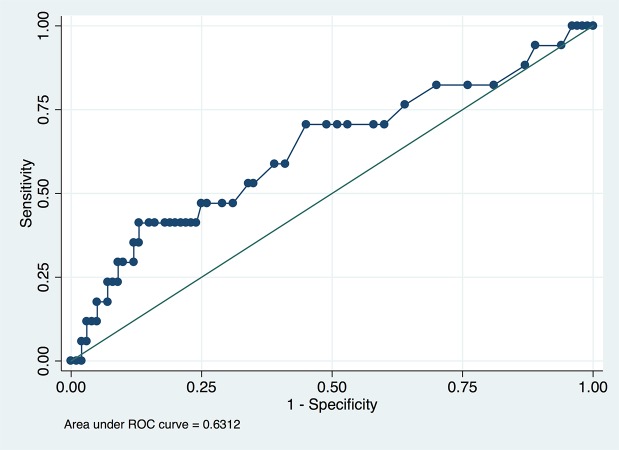
Receiver operating characteristic curve of the initial lactate to predict AKI development. The area under curve was 0.63.

The diagnostic performance of initial lactate in predicting AKI is shown in Table 6. With increasing cutoff points from 1.1 to 7.4 mmol/l, the sensitivity decreased from 94.12% to 11.76%, whereas the specificity increased from 6.00% to 95.00%. The area under receiver operating characterisc curve was 0.63 (95% CI: 0.47–0.79, Fig. 5). The best sensitivity and specificity were 41.2% and 87.0% at the cutoff point of 4.4 mmol/l. Table 7 shows different diagnostic performances of serum lactate in predicting AKI at different time points. It was a tradeoff between timeliness and accuracy. That is, while lactate measured early may be timely enough but lacks accuracy, lactate measured late may be accurate but too late.

**Table 6:** Diagnostic performance of initial lactate in predicting AKI.

Cutoff values	Sensitivity (%)	Specificity (%)	LR+	LR−
>1.1	94.12	6.00	1.0013	0.9804
>2.1	70.59	49.00	1.3841	0.6002
>3.1	47.06	75.00	1.8824	0.7059
>4.4	41.18	87.00	3.1674	0.6761
>5.0	29.41	90.00	2.9412	0.7843
>7.1	11.76	95.00	2.3529	0.9288

Abbreviations: LR+: positive likelihood ratio; LR-: negative likelihood ratio. Area under ROC was 0.63 (95% CI: 0.47–0.79).

**Table 7:** Diagnostic performance of lactate measured at different time points.

Hours after ICU entry (95% CI)	Area under ROC (95% CI)
0	0.63 (0.47–0.79)
4 (1–10.3)	0.66 (0.56–0.73)
8.4 (2.5–19.6)	0.73 (0.64–0.81)
14.5 (4.8–32.4)	0.69 (0.60–0.78)
20.9 (7.9–29.3)	0.76 (0.66–0.83)
24.8 (8.6–44.4)	0.70 (0.58–0.80)
28.1 (10.9–45.6)	0.63 (0.48–0.78)
28.8 (13–55.7)	0.88 (0.68–0.99)

## Discussion

The study demonstrates that L_ln_ is independently associated with postoperative AKI after CPB. Although the two-term fractional polynomial model with the power 3 and 3 is the best fitted model, it is not significantly different from the linear model. Serum lactate levels are influenced by various factors during and after CPB. During CPB, hyperlactatemia may come from: 1) lactate’s ringer solution administration (priming pump), 2) low-perfusion pressure due to distributive shock, 3) type-B hyperlactatemia (frequently associated with hyperglycemia). Differently, most of the causes of hyperlactatemia due to inadequate oxygen supply can be identified after the CPB phase, including the left/right pump dysfunction and/or distributive shock. Pump dysfunction was evaluated by the use of inotrope and distributive shock was represented by the use of vasopressors in our study. Unfortunately, both of these variables were not significantly different between AKI and non-AKI groups, probably due to limited sample size and lack of statistical power.

Our study is consistent with the study by Lopez-Delgado JC and colleagues,[31] in which they demonstrated that arterial lactate 24 hours after admission is an independent risk factor for the development of AKI with an odds ratio of 1.810 (95% CI: 1.300 to 2.015). However, the logistic regression model was not sufficiently checked for its model fit and discrimination, and also potential interaction terms were not explored. Therefore, although Lopez-Delgado’s study included a large sample size, our results supplemented that one by building multivariable model with rigorous methodology. By using such rigorous methodology, our model has an excellent discrimination power as represented by an area under ROC of 0.84. In another observational study, Hajjar LA and coworkers[32] found that higher lactate was associated with postoperative major complications, but AKI was not analyzed separately from other major complication events. In pediatric patients undergoing CPB due to congenital heart disease, a lactate level of more than 4.8 mmol/l had an odds ratio of 16.9 for the need of peritoneal dialysis.[33] In contrast to our study, they used the need for peritoneal dialysis as a measure of renal outcome. However, in our study cohort no patients developed so severe kidney injury that renal replacement therapy was initiated (all AKI patients were in RIFLE-R and RIFLE-I stages). Furthermore, previous studies treated lactate as a point measurement, and its accumulation over time has not been considered. In contrast, we utilized lactate load as a measurement of hypoperfusion accumulated with time, under the assumption that the effect of global hypoperfusion on AKI is time dependent.

Duration of CPB and cross-clamp time have been shown to be directly associated with postoperative AKI. In a systematic review and meta-analysis, Kumar and coworkers showed that the mean durations of CPB were statistically longer in the AKI group as compared with non-AKI group. The mean differences in the duration of CPB between the two groups were 23.18 minutes with the random-effects model and 25.65 minutes with the fixed-effects model.[34] Also, in a multivariable regression model, Parolari A and colleagues found that cross-clamp time was an independent risk factor for AKI.[5] Our results are consistent with those previous studies in bivariate analysis that Patients with longer duration of CPB were more likely to develop AKI (82.9±33.8 vs 66.6±26.4 minutes, p = 0.026), and longer cross-clamp time was associated with increased risk of AKI (57.8±28.3 vs 46.2±20.9 minutes, p = 0.049). However, this relationship did not hold true in multivariable model after stepwise covariate selection, and both duration of CPB and cross-clamp time were excluded in the main effect model. CPB is associated with remarkable changes in hemodynamic pattern as compared with the physiological one. Particularly, CPB provides nonpulsatile flow that may influence tissue perfusion and oxygen delivery. There is a few evidence showing that prolonged CPB is associated with increases in serum lactate levels.[35]

Fluid balance is another important risk factor for the development of AKI.[36] Our study shows that more positive fluid balance within the first 24 hours is associated with increased risk of AKI, and this relationship is robust in multivariable model. The relationships between fluid balance and AKI are important issues that have to be considered under different aspects. Firstly, a positive fluid balance may be related with impaired cardio-circulatory function (left, right or biventricular dysfunction, distributive shock, capillary leak syndrome) that may lead to fluid over-load (or higher fluids administration) with the attempt to compensate for tissue hypoperfusion. In this case the relationship with AKI could be related with low-DO2 state or compromised microvascular flow. In our study, cardiac function was considered by whether a patient is on inotrope or not. The result showed that the number of patients with inotrope support was not statistically different. Secondly, inappropriate fluid administration with fluid overload may have led to excessive increase in central venous pressure and venous congestion. Venous congestion has been identified as a risk factor for AKI development. Perioperative fluid therapy is still a matter of great debate [36]. Observational studies on critically ill patients suggest that fluid accumulation and tissue edema are associated with the development of AKI [37], progression to more severe AKI and increased mortality in patients with AKI [38]. In a systematic review and meta-analysis, goal-directed conservative fluid management is proven to be effective in reducing the incidence of AKI [39]. In patients undergoing cardiovascular surgery, the adjusted OR for postoperative AKI for the highest versus lowest quartile of fluid balance was 4.98 (95%CI:1.38–24.10, P = 0.046).[40] Early postoperative fluid overload defined as >5% increase of the body weight was associated with development of AKI, and more often preceded it than followed it.[41] The temporal sequence observed in clinical practice is fundamental to the establishment of causal relationship between fluid balance and AKI. Due to the close correlation of fluid balance and AKI incidence, we hypothesized there would be an interaction between fluid balance and lactate for AKI, that is, the effect of hyperlactatemia on AKI may be altered by different fluid management strategy. However, we failed to identify such an interaction, probably attributable to limited number of events in the present study.

Several limitations in the present study should be acknowledged. Firstly, this is an observational study that is subject to potential bias. For instance, selection bias can be resulted from such design that only patients with less comorbidity are enrolled for cardiac surgery. Because our institution is not the center of cardiac surgery and more complicated patients are generally referred to other hospitals. Such selection bias will lead to limited generalization of current conclusions. Secondly, because of the observational nature, the measurement of lactate was not protocolized in terms of frequency and the total number of measurements. Measurement bias may be introduced in that more critically ill patients were ordered more lactate measurements. We tried to limit the impact of such bias by normalizing lactate load to time. Thirdly, although lactate was found to be independently associated with AKI, the causal relationship cannot be established. Lactate was only a post-operative marker of hemodynamic status, given that other postoperative parameters were lacking. Controlled trials exploring strategies to resolve hyperlactatemia should be performed to address this issue. Lastly and the most importantly, the number of AKI cases was low in the study, which significantly limited the reliability of our model. The result of our study can only be hypothesis generating and requires further confirmation.

In aggregate, our study demonstrates that L_ln_ is independently associated with postoperative AKI in patients undergoing CPB. There is no significant interaction with early postoperative fluid balance.
